# Visit Experience and Fulfillment of Care Needs in Primary Care Differs for Video Visits Compared to In-person and Chat Visits

**DOI:** 10.1007/s11606-024-08781-z

**Published:** 2024-05-17

**Authors:** Daniel Söderberg, Stephanie E. Bonn, Linnea Sjöblom, Anna Dahlgren, Irene Muli, Isis Amer-Wåhlin, Bo C. Bertilson, Nasim Farrokhnia, Helena Hvitfeldt, Marina Taloyan, Maria Hägglund, Ylva Trolle Lagerros

**Affiliations:** 1 Division of Clinical Epidemiology, Department of Medicine (Solna), Karolinska Institutet, Karolinska University Hospital, Solna, Stockholm Sweden; 2 Participatory eHealth and Health Data Research Group, Department of Women’s and Children’s Health, Uppsala University, Uppsala, Sweden; 3 Medical Management Centre, Department of Learning, Informatics, Management and Ethics, Karolinska Institute, Solna, Sweden; 4 Digital Health Unit, Research Institute of Sweden, Stockholm, Sweden; 5 Division of Family Medicine and Primary Care, Department of Neurobiology, Care Sciences and Society, Karolinska Institutet, Solna, Sweden; 6 Academic Primary Health Care Center, Stockholm Health Care Services, Region Stockholm, Stockholm, Sweden; 7 Department of Clinical Science and Education, Södersjukhuset, Karolinska Institutet, Stockholm, Sweden; 8 Norrtälje Hospital, Vårdbolaget Tiohundra, Stockholm, Sweden; 9 Center for Obesity, Academic Specialist Center, Stockholm Health Care Services, Stockholm, Sweden

**Keywords:** telemedicine, general medicine, patient experience, quality of care

## Abstract

**Background:**

There is a lack of research comparing patient experience and to what extent patients’ care needs are fulfilled in telemedicine compared to in-person care.

**Objective:**

To investigate if patient experience and fulfillment of care needs differ between video and chat visits with direct to consumer telemedicine providers compared to in-person visits.

**Design:**

Cross-sectional study.

**Participants:**

Adults visiting a primary care physician in person or via chat or video in Region Stockholm, Sweden, October 2020–May 2021.

**Main Measures:**

Patient-reported visit experience and fulfillment of care needs.

**Key Results:**

The sample included 3315 patients who had an in-person (1950), video (844), or chat (521) visit. Response rates were 42% for in-person visitors and 41% for telemedicine visitors. Patients were 18–97 years old, mean age of 51 years, and 66% were female. In-person visitors reported the most positive patient experience (“To a very high degree” or “Yes, completely”) for being listened to (64%), being treated with care (64%), and feeling trust and confidence in the health care professional (76%). Chat visitors reported the most positive patient experience for being given enough time (61%) and having care needs fulfilled during the care visit (76%). Video visitors had the largest proportion of respondents choosing “To a very low degree” or “No, not at all” for all visit experience measures. There were statistically significant differences in the distribution of visit experiences between in-person, video, and chat visits for all visit experience measures (*P* < 0.001).

**Conclusions:**

Video visits were associated with a more negative visit experience and lower fulfillment of care needs than in-person visits. Chat visits were associated with a similar patient experience and fulfillment of care needs as in-person visits. Chat visits may be a viable alternative to in-person visits for selected patients.

**Supplementary Information:**

The online version contains supplementary material available at 10.1007/s11606-024-08781-z.

## INTRODUCTION

During the last decade, direct to consumer (DTC) telemedicine through digital platforms for video and chat communication has increased in primary health care.^1,2^ The use accelerated further during the COVID-19 pandemic.^3,4^

The rapid development of DTC telemedicine in primary care has started a discussion about to what extent telemedicine is a valid substitute for in-person visits at primary health care centers. DTC telemedicine has been shown to produce lower clinical quality and has been associated with more follow-up visits compared to in-person care.^2,5^ Telemedicine visits with the patient’s regular primary health care provider may however not be associated with these outcomes,^6^ and patients using DTC telemedicine find the service satisfying.^7–9^ DTC telemedicine visits can be both synchronous (video visits, telephone contacts, or live chats) or asynchronous (chats where messages are stored while waiting for response).^10^

Few studies investigating the experience of patients using telemedicine have made comparisons to in-person care.^11^ This makes it difficult to draw conclusions about the validity of telemedicine as a substitute for in-person care. In their review of patient satisfaction and experience with telemedicine during the COVID-19 pandemic, Aashima^12^ only found two studies comparing experience or satisfaction between telemedicine and in-person care: Ramaswamy^13^ reported that patient satisfaction was higher for patients using telemedicine compared to in-person care, while Itamura^14^ reported opposite results. However, neither of the two studies was conducted in a primary health care setting.

In this study, we aimed to investigate if patient experience and fulfillment of care needs differed between video and chat visits compared to in-person visits with primary health care physicians.

## METHODS

### Design

In this cross-sectional study, we surveyed patients using in-person or DTC telemedicine primary health care during October 2020–May 2021. Participants were consecutively recruited from six primary health care centers in Region Stockholm, Sweden, and four providers of telemedicine primary health care in Sweden. Participants answered the questionnaire either on paper or via a secure online survey system.

All participants provided informed consent. The study was approved by the Swedish Ethical Review Authority (Dnr: 2020–00860, amendments 2020–06506 and 2021–04602). The guideline for Strengthening the Reporting of Observational Studies in Epidemiology (STROBE) was followed.

### Setting

The Swedish health care system is publicly funded. Patients pay a small fee for each visit (in Region Stockholm, 200 Swedish krona (SEK), equivalent to about 20 USD), up to an annual total of 1150 SEK (110 USD), after which all health care is free of charge. Most patients have a regular primary care provider, but can seek health care with any primary health care provider in the country, including any DTC telemedicine provider. Telemedicine providers offer lower or no patient fees. Some health care centers also offer telemedicine visits for listed patients, although these services were not included in this study.

The telemedicine providers were well-known, three private and one public, with a number of physicians employed. They used different technological platforms for their services. Patients were initially triaged using a questionnaire or chat and could thereafter be referred to in-person care at a primary care center or elsewhere, if deemed inappropriate for telemedicine care. One provider only offered video visits, while the other providers used chat, video, or a combination thereof, depending on the complaint of the patient. Video visits consisted of synchronous audio and video communications, while chat visits included both synchronous and asynchronous text communication.

All providers had access to patient records through a national health information exchange infrastructure, through which the majority of Swedish electronic health records can be accessed.

### Recruitment

Inclusion criteria were being ≥ 18 years of age and having visited a participating care provider during the study period either as a patient, as a guardian of a minor, or as an informal caregiver of someone seeking care. Exclusion criteria were being unable to give informed consent. Patients whose visits were due to COVID-19 were excluded in order to minimize the impact of the pandemic on the results.

At primary health care centers, patients were recruited by study personnel in connection with an in-person visit. Patients were given a brief description of the study, and if they expressed interest in participating, they were provided the full written study information. If the patient was < 18 years old, or was unable to participate due to, e.g., cognitive impairment, the guardian or an informal caregiver accompanying the patient was asked to participate on behalf of the patient. Only one questionnaire was distributed per completed visit. Participants could choose to fill out a printed questionnaire directly or take it home and return it in a prepaid envelope. Participants who preferred to answer online provided study personnel with their email address and received an emasil with a link to the questionnaire the same day. At one center, health care professionals handled the recruitment due to COVID-19 restrictions.

Eligible telemedicine participants were invited through a message either through the digital provider’s platform or via email within 24 h of their contact. The invitation included a brief description of the study and a link to the online questionnaire which also contained the full study information. Participants could request to receive a printed version of the questionnaire via mail, although only one respondent chose this option.

The screening process differed slightly between telemedicine providers due to differences in their platforms’ ability to identify patients with the specified inclusion and exclusion criteria. One provider was able to identify the patients eligible for participation and invited only those to participate. Another provider could filter patients on cause of visit (thus filtering out patients who sought care for reasons related to COVID-19) and combined this with a few questions to be able to invite patients who fulfilled eligibility criteria. Other providers could screen for some of the eligibility criteria, but specified remaining criteria in the invitation message.

Since the questionnaire also included questions about inclusion criteria, respondents who were incorrectly recruited to the study were excluded from the analysis.

### Analysis Population

In total, 4388 patients responded to the questionnaire. Response rates were 42% for in-person visitors at primary health care centers, based on the number of distributed questionnaires, and 41% for telemedicine visitors, based on the number of patients who clicked on the link in the invitation message. The number of patients who were screened for participation and invited was not recorded.

In the analysis, we included data from in-person, video, or chat visits to physicians by patients living in Region Stockholm. Thus, we excluded 253 respondents who had a different type of visit and 531 respondents who had met with another health care professional (e.g., nurse or psychologist). Furthermore, we excluded 286 patients due to residency outside of Region Stockholm, as these patients predominantly used telemedicine and could skew the analysis. Finally, 3 respondents were excluded as they were < 18 years, but had filled out the questionnaire themselves rather than having it be completed by a guardian.

### Questionnaire

The questionnaire included demographics, self-rated health, worry at time of scheduling the appointment, and internet habits (Supplementary Table 1). To assess visit experience and fulfillment of care needs, we included questions used in the General Practice Patient Survey, a well-established survey used to evaluate primary health care in the UK.^15–17^ The questionnaire was evaluated through cognitive interviews, during which 7 informants commented on the written instructions, the questionnaire’s structure, the questions, and their wording while filling it out. The process led to minor clarifications, for example, the question regarding the respondent’s health was clarified to emphasize that it referred to the respondent’s general health and not their health at the time of seeking care. We also clarified instructions such as what question to continue to, when answering yes or no.

Patient experience was evaluated using four questions asking respondents whether they had been listened to, been treated with care, been given enough time, and felt trust for the health care professional they met during their care visit. Fulfillment of care needs was evaluated with a separate question. Questions about whether respondents had been listened to, treated with care, and been given enough time during the care visit were answered on a 5-point scale ranging from “To a very low degree” to “To a very high degree.” Questions about feeling trust and confidence in the health care professional they met and whether respondents had their care needs fulfilled during the visit were answered on a 4-point scale ranging from “No, not at all” to “Yes, completely.”

Participants were categorized into four age groups based on quartiles: 18–37 years, 38–50 years, 51–63 years, and ≥ 64 years. Education was categorized into ≤ 12 years or > 12 years. Participants were categorized as working, retired, or other (e.g., studying, unemployed, or on sick leave). For self-rated health, which had 5 response alternatives ranging from “very bad” to “very good,” we combined the two response alternatives “bad” and “very bad” into one category as less than 1% rated their health as very bad. For internet use, participants were categorized as daily internet users or not. For length of visits, we combined the categories 20–30 min and > 30 min when used as a covariate in the regression models, as only 0.6% of video visits were > 30 min.

### Power

Previous studies using questions from the General Practice Patient Survey found 80–90% to be satisfied/very satisfied with interpersonal aspects, such as experiencing being listened to.^16^ Assuming that 90% of the in-person visits were satisfied or very satisfied, 1400 subjects would be needed to detect a difference of 5% compared to chat or video visits.

### Statistical Analysis

Statistics were reported as *n* (%) for all categorical variables. Differences in characteristics between types of care were compared using chi^2^-tests. *P*-values < 0.05 were considered statistically significant. Data was analyzed using Stata 15.1 (Stata Corporation, College Station, TX, USA).

## RESULTS

### Demographics and Visit Characteristics

Our final sample included 3315 respondents, who were 18–97 years old, with a mean age of 51 years, and 33% males (Table 1). Of these, 1950 (59%) visited a physician at a primary health care center in-person (in-person visitors), 844 (25%) communicated with a primary care physician using video (video visitors), and 521 (16%) chatted with a primary care physician (chat visitors). A small proportion, 242 (7%), had visited the care provider as a guardian of children under 18 years of age. It was more common among video visitors (70%) and chat visitors (63%) than among in-person visitors (55%) to have an education > 12 years. Video and chat visitors were also working to a larger extent (80% and 78%, respectively) than in-person visitors (57%). Around 27% of both video and chat visitors reported very good health, compared to 17% for in-person visitors. Between 6 and 9% of respondents reported bad or very bad health in all types of care. Chat visitors were less worried than both in-person and video visitors when scheduling their appointment (55% not worried at all compared to 27.6% for in-person visits and 32% for video visits). In-person visits were longer than video and chat visits; 87% of in-person visits were ≥ 10 min, compared to 32% of video visits and 39% of chat visits.

**Table 1:** Characteristics of Participants and Visit Length by Type of Visit (In-person, Video, Chat)

	Total *N* = 3315	In-person *N* = 1950	Video *N* = 844	Chat *N* = 521	*P*-value*
	*N* (%)	*N* (%)	*N* (%)	*N* (%)	
Age group (years)					< 0.001
18–37	816 (24.6)	379 (19.5)	222 (26.3)	215 (41.3)	
38–50	887 (26.8)	386 (19.8)	329 (39.0)	172 (33.0)	
51–64	783 (23.6)	476 (24.4)	210 (24.9)	97 (18.6)	
≥ 64	827 (25.0)	707 (36.3)	83 (9.8)	37 (7.1)	
Guardian of child					< 0.001
Yes	242 (7.3)	95 (4.9)	102 (12.1)	45 (8.6)	
Gender					< 0.001
Female	2193 (66.4)	1228 (63.2)	577 (68.9)	388 (74.5)	
Male	1092 (33.1)	708 (36.4)	254 (30.3)	130 (25.0)	
Other/unknown	16 (0.5)	7 (0.4)	6 (0.7)	3 (0.6)	
Education					< 0.001
≤ 12 years	1315 (40.1)	875 (45.4)	249 (29.7)	191 (36.9)	
> 12 years	1966 (59.9)	1051 (54.6)	588 (70.3)	327 (63.1)	
Occupation					< 0.001
Working	2168 (66.0)	1093 (56.7)	671 (80.0)	404 (78.1)	
Retired	741 (22.6)	640 (33.2)	66 (7.9)	35 (6.8)	
Other	376 (11.4)	196 (10.2)	102 (12.2)	78 (15.1)	
Self-rated health					< 0.001
Very good	696 (21.1)	323 (16.7)	231 (27.4)	142 (27.3)	
Good	1678 (50.8)	1005 (51.8)	418 (49.5)	255 (49.0)	
Average	659 (20.0)	437 (22.5)	137 (16.2)	85 (16.3)	
Bad or very bad	270 (8.2)	174 (9.0)	58 (6.9)	38 (7.3)	
Worry when scheduling					< 0.001
Very worried	116 (4.5)	65 (4.6)	37 (5.0)	14 (3.1)	
Somewhat worried	546 (21.1)	336 (23.9)	154 (21.0)	56 (12.5)	
A little worried	1055 (40.8)	616 (43.9)	307 (41.9)	132 (29.5)	
Not worried at all	868 (33.6)	387 (27.6)	235 (32.1)	246 (54.9)	
Length of care visit					< 0.001
< 10 min	1096 (34.2)	251 (13.1)	546 (67.7)	299 (61.1)	
10–20 min	1196 (37.3)	838 (43.9)	242 (30.0)	116 (23.7)	
20–30 min	625 (19.5)	572 (29.9)	14 (1.7)	39 (8.0)	
> 30 min	289 (9.0)	249 (13.0)	5 (0.6)	35 (7.2)	

^*^Chi^2^-test of distribution between the types of care

### Visit Experience

**Figure 1:**
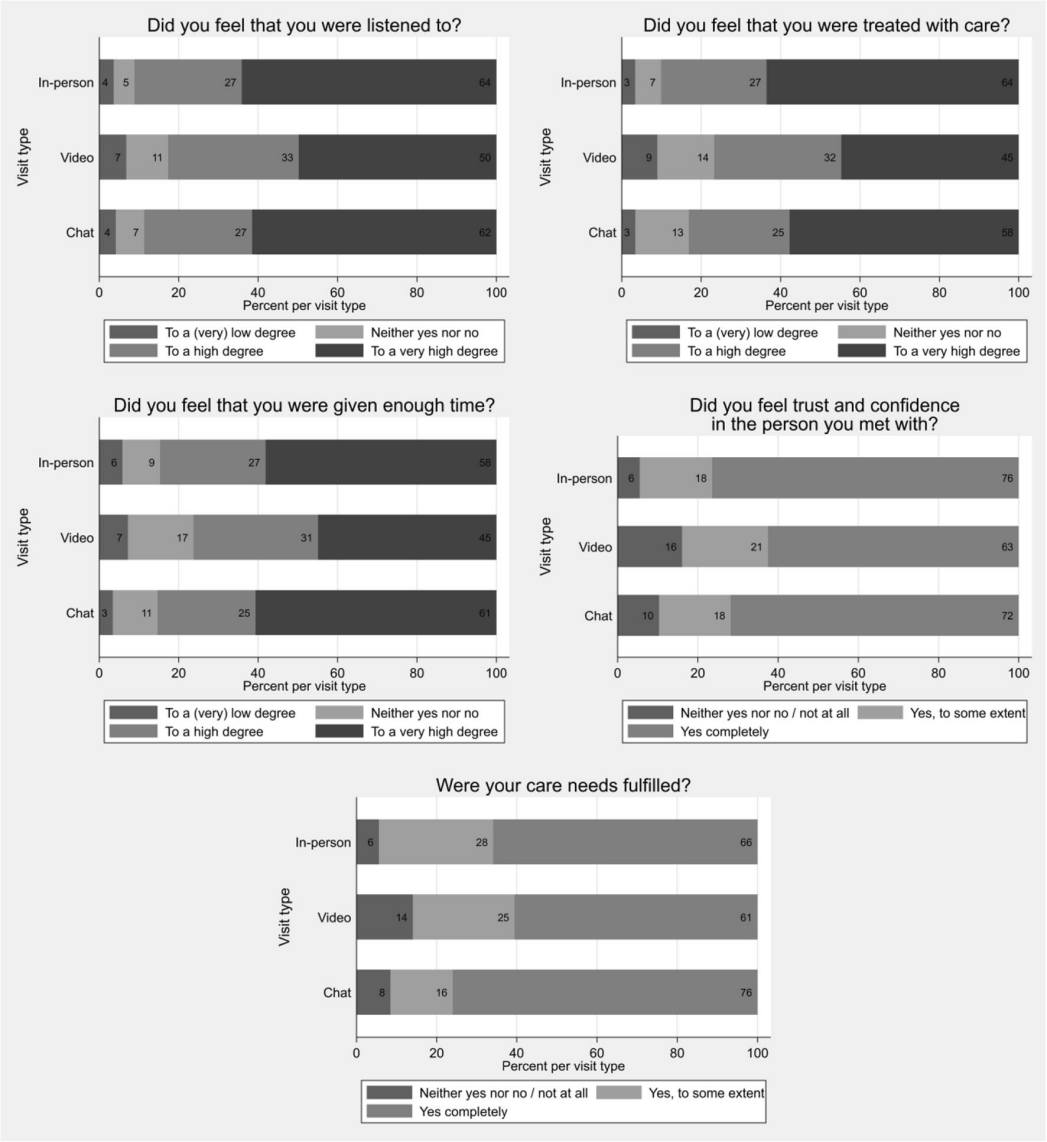
Patient-reported experience and fulfillment of care needs during the care visit, divided by type of visit (in-person, video, or chat).

In-person visitors reported the most positive patient experience (“To a very high degree” or “Yes, completely”) for being listened to (64%), being treated with care (64%), and feeling trust and confidence in the health care professional (76%) (Fig. 1). Chat visitors reported the most positive patient experience for being given enough time (61%) and having care needs fulfilled during the care visit (76%) (Fig. 1, for details see Supplementary Table 2). More than 50% of in-person and chat visitors responded “To a very high degree” or “Yes, completely” for all visit experience measures. Video visitors, on the other hand, had the smallest proportion of respondents choosing “To a very high degree” or “Yes, completely” and the largest proportion of respondents choosing “To a very low degree” or “No, not at all” for all visit experience measures. There were statistically significant differences in the distribution of visit experiences between in-person, video, and chat visits for all visit experience measures (*P* < 0.001).

## DISCUSSION

Our study showed that patients using chat experienced fulfillment of care needs comparable to that of in-person visits, while video visitors to a lower extent experienced that they had been listened to, treated with care, and given enough time; had felt trust; and had their care needs fulfilled during their visit compared to in-person care.

These findings suggest that different types of telemedicine, such as video or chat contacts, have distinct features that make them suitable for different patient groups and circumstances.

Previous studies have explored the experience and satisfaction of patients using telemedicine in primary care. The majority of these studies reported high levels of patient satisfaction.^7–9, 18^ In a review of telemedicine services, Kruse et al.^11^ found the use of telemedicine to be associated with high patient satisfaction and ease of use, as well as improved health outcomes such as decreased hospital admissions. Contrarily, patients having video visits in our study reported on average worse experience and lower fulfillment of care needs compared to in-person visits. However, in the review by Kruse et al., the majority of included studies surveyed a well-defined patient group with a specific type of telemedicine intervention. In our study, the visits were patient-initiated and included various patient groups. This may reduce the usability of video visits as patients may require physical examinations to a higher degree. Other studies have included specialist care^19^ while our study only included primary care, which constitutes a more unselected patient cohort where physical encounters may be more important to identify and triage causes of patient symptoms. Finally, patients’ regular primary care provider could potentially have provided a better video experience than a provider unassociated with the regular provider.

While some studies indicate that both experience and satisfaction may be linked to clinical outcomes of care^20,21^ — a more traditional measure of care quality — many authors argue that patient experience and satisfaction are in fact independent dimensions of care quality which should be given as much attention as clinical outcomes.^22–24^ Patient satisfaction has been criticized for being influenced by patient expectations, while patient experience, as used in our study, asks patients what happened and how they experienced it, rather than asking them to make a value judgement about their experience. As such, this measure is considered more objective and also better for comparison between caregivers.^23, 25^

Previous research on the association between visit length and quality of care, including patient experience, has yielded mixed results. Swanson and colleagues^26^ found that shorter (15 min) primary care appointments were associated with less hospitalizations and consumption of additional health care within a week of the visit, compared to longer appointments (> 30 min). Although differences could be due to selection bias, their conclusion was that for certain patient groups, shorter visits may still be a suitable and safe option. In an experimental study using standardized patients, Elmore and colleagues^27^ found no association between visit length and patient experience. Wilson and colleagues^28^ concluded that the evidence for interventions targeting visit length on patient outcomes is too scarce to draw any firm conclusions.

In our study, patients with video visits had the shortest self-reported visit length, followed by chat visits. However, visit length is a difficult measure to compare between different visit types, as an in-person visit could include, for example, a physical examination. The visit length of a chat visit can also be measured differently depending on whether the chat is synchronous or asynchronous. Herein, we only have access to patients’ self-reported visit length, making this measure somewhat uncertain.

### Strengths and Limitations

A strength of this study is that we included three out of the four major private providers of telemedicine primary care in Sweden, together serving around 66% of the market in 2020,^29^ as well as the sole public provider of telemedicine primary care in Region Stockholm. However, patient experience and quality may differ between providers of care. It would be informative to reproduce the study also including smaller telemedicine providers as well as public regional telemedicine providers.

Another strength of the study is the use of questions on visit experience derived from an established questionnaire designed for evaluation of primary care.^15^ Although the translation of these questions was not validated separately in the context of this study, the translation was tested as part of the psychometrical evaluation of the questionnaire.

The study took place during the COVID-19 pandemic and patient experience and preferences may have been affected by this circumstance. A challenge with comparing telemedicine to in-person care is that user groups may not be comparable between the care types^30^. Indeed, many studies have found that there are differences between patients using in-person care and telemedicine.^1, 18, 31^ As such, selection bias may account for some of the differences in outcomes. One of the limitations of this study is that we investigated differences in experiences of in-person visits and telehealth in general, without taking into consideration differences in experiences of telemedicine visits with the same physician in the same organization as the patient’s regular primary health care provider.

Video and chat visitors may also have different reasons for care visits, and hence have different care needs. In our setting, patients were able to choose between in-person or telemedicine care. However, the telemedicine providers had different communication strategies: two telemedicine providers almost only communicated with patients using video, while the remaining two predominantly communicated with patients using chat. The choice between video or chat communication was made by the telemedicine provider, and it is unclear if patients were aware of these differences between the telemedicine providers. Furthermore, there were differences in the recruitment processes and responding to the survey on paper vs. digitally might have affected the sampling and results. Low response rates could also have resulted in response bias.

## CONCLUSIONS

Chat visits in primary health care delivered patient experience and fulfillment of care needs similar to those of in-person visits, while patients communicating via video reported more negative visit experience and lower fulfillment of care needs. While chat visits can be an alternative to in-person visits, reasons for the lower performance of video visits must be investigated.

## Supplementary Information

Below is the link to the electronic supplementary material.

Supplementary file1 (DOCX 28 KB)

## Data Availability

The datasets generated during and analyzed during the current study are not publicly available due to ethical restrictions but are available from the corresponding author on reasonable request.
