# Supplementary material for: Visit Experience and Fulfillment of Care Needs in Primary Care Differs for Video Visits Compared to In-person and Chat Visits
Source: J Gen Intern Med. 2024 May 17;39(15):2881–7. doi: 10.1007/s11606-024-08781-z (PMC11576744; doi:10.1007/s11606-024-08781-z)
Supplement: Supplementary file 1 — Supplementary file1 (DOCX 28 KB) [file 11606_2024_8781_MOESM1_ESM.docx]

APPENDICES

| **Supplementary Table 1: Questions used in questionnaire** | | |
| --- | --- | --- |
| **Question** | **Type** | **Options (if applicable)** |
| Who was this care visit about? | Single choice | Myself  My child (under 18 years of age)  Someone else (such as a relative, child over 18 years of age) |
| State your gender: | Single choice | Male  Female  Other/don’t want to answer |
| What year were you born? | Free text |  |
| Which is you highest finished education? | Single choice | Elementary school  High school (2 years) or vocational school  High school (3-4 years)  Folk high school  University less than 3 years  University 3 years or more |
| Which is your main occupation? | Multiple choice | Working full time  Working part time  Studying  Unemployed  Sick leave  Retired  Parental leave  Don’t know/don’t want to answer |
| How do you rate your general health? | Single choice | Very good  Good  Neither good nor bad  Bad  Very bad |
| Which of the following do you have access to? | Multiple choice | Mobile phone without apps  Mobile phone with apps (smartphone)  Computer  Tablet  None of the above |
| How often do you use internet with the following device?   - Mobile phone - Computer - Tablet | Single choice (for each option) | Daily  Every week  Every month  Occasionally  Never |
| During this care visit, did you feel that you…  …were listened to?  …were treated with care?  …were given enough time? | Single choice (for each option) | To a very high degree  To a high degree  Neither a high nor a low degree  To a low degree  To a very low degree |
| During this care visit, did you feel confidence and trust in the person you met with? | Single choice | Yes, completely  Yes, to some extent  Neither yes nor no  No, not at all |
| Were your care needs fulfilled during this care visit? | Single choice | Yes, completely  Yes, to some extent  Neither yes nor no  No, not at all |

| **Supplementary Table 2: Patient experience grouped by type of visit (in-person, video, chat).** | | | | | |
| --- | --- | --- | --- | --- | --- |
|  | **Total**  **N=3315** | **In-person**  **N=1950** | **Video**  **N=844** | **Chat**  **N=521** | **P-value*** |
|  | N(%) | N(%) | N(%) | N(%) |  |
| During this care visit, did you feel that you were listened to? | | | | | <0.001 |
| To a very high degree | 1953(60.1) | 1233(64.1) | 410(49.8) | 310(61.5) |  |
| To a high degree | 928(28.5) | 520(27.0) | 271(32.9) | 137(27.2) |  |
| Neither yes nor no | 223(6.9) | 100(5.2) | 87(10.6) | 36(7.1) |  |
| To a low degree | 92(2.8) | 41(2.1) | 33(4.0) | 18(3.6) |  |
| To a very low degree | 55(1.7) | 29(1.5) | 23(2.8) | 3(0.6) |  |
| During this care visit, did you feel that you were treated with care? | | | | | <0.001 |
| To a very high degree | 1851(57.8) | 1199(63.5) | 365(44.7) | 287(57.7) |  |
| To a high degree | 888(27.8) | 501(26.6) | 261(32.0) | 126(25.4) |  |
| Neither yes nor no | 307(9.6) | 123(6.5) | 117(14.3) | 67(13.5) |  |
| To a low degree | 88(2.8) | 35(1.9) | 43(5.3) | 10(2.0) |  |
| To a very low degree | 66(2.1) | 29(1.5) | 30(3.7) | 7(1.4) |  |
| During this care visit, did you feel that you were given enough time? | | | | | <0.001 |
| To a very high degree | 1763(55.1) | 1094(58.1) | 367(44.9) | 302(60.6) |  |
| To a high degree | 880(27.5) | 501(26.6) | 256(31.3) | 123(24.7) |  |
| Neither yes nor no | 369(11.5) | 178(9.5) | 135(16.5) | 56(11.2) |  |
| To a low degree | 123(3.8) | 70(3.7) | 41(5.0) | 12(2.4) |  |
| To a very low degree | 63(2.0) | 40(2.1) | 18(2.2) | 5(1.0) |  |
| During this care visit, did you feel trust and confidence in the person you met with? | | | | | <0.001 |
| Yes, completely | 2345(72.2) | 1469(76.4) | 514(62.5) | 362(71.8) |  |
| Yes, to some extent | 613(18.9) | 347(18.1) | 176(21.4) | 90(17.9) |  |
| Neither yes nor no | 165(5.1) | 60(3.1) | 68(8.3) | 37(7.3) |  |
| Not at all | 125(3.8) | 46(2.4) | 64(7.8) | 15(3.0) |  |
| Were your care needs fulfilled during this care visit? | | | | | <0.001 |
| Yes, completely | 2127(66.1) | 1262(65.9) | 488(60.5) | 377(76.0) |  |
| Yes, to some extent | 827(25.7) | 545(28.5) | 205(25.4) | 77(15.5) |  |
| Neither yes nor no | 114(3.5) | 60(3.1) | 35(4.3) | 19(3.8) |  |
| Not at all | 148(4.6) | 47(2.5) | 78(9.7) | 23(4.6) |  |
| *Chi2-test of distribution between the types of care. | | | | | |
